# The PML hub: An emerging actor of leukemia therapies

**DOI:** 10.1084/jem.20221213

**Published:** 2023-06-29

**Authors:** Domitille Rérolle, Hugues de Thé

**Affiliations:** 1 Center for Interdisciplinary Research in Biology, Collège de France, Inserm, PSL Research University, Paris, France; 2 Université Paris Cité, Inserm U944, CNRS, GenCellDis, Institut de Recherche Saint-Louis, Paris, France; 3 Chaire d'Oncologie Cellulaire et Moléculaire, Collège de France, Paris, France; 4 Service d'Hématologie Biologique, Assistance Publique-Hôpitaux de Paris, Hôpital St. Louis, Paris, France

## Abstract

PML assembles into nuclear domains that have attracted considerable attention from cell and cancer biologists. Upon stress, PML nuclear bodies modulate sumoylation and other post-translational modifications, providing an integrated molecular framework for the multiple roles of PML in apoptosis, senescence, or metabolism. PML is both a sensor and an effector of oxidative stress. Emerging data has demonstrated its key role in promoting therapy response in several hematological malignancies. While these membrane-less nuclear hubs can enforce efficient cancer cell clearance, their downstream pathways deserve better characterization. PML NBs are druggable and their known modulators may have broader clinical utilities than initially thought.

## Teaser

PML nuclear bodies are stress-responsive domains involved in senescence, apoptosis, or metabolism. PML is required for response or even cure of some hematological malignancies. The authors review how PML participates in cancer cell clearance and highlight its druggable aspect.

## Introduction

Promyelocytic leukemia (PML) was discovered in the context of acute promyelocytic leukemia (APL), of which *PML::RARA* oncogenic fusion is the primary, if not sole, driver (de Thé et al., 2017; Lehmann-Che et al., 2018). Arsenic trioxide (ATO), a miracle APL therapy (Zhu et al., 2019), targets both PML and PML::RARA. PML is required to cure human or murine APLs. Vibrant PML research has developed in multiple labs, focused on cell biology, virology, biochemistry, and roles of PML as a key modulator of stress response and tumor suppression (Gamell et al., 2014). Here, we will briefly summarize these aspects to focus on the emerging role of PML as an under-recognized hub enforcing response of hematological malignancies to different therapies.

## PML nuclear bodies are dynamic and stress-sensitive structures

**Figure 1.**
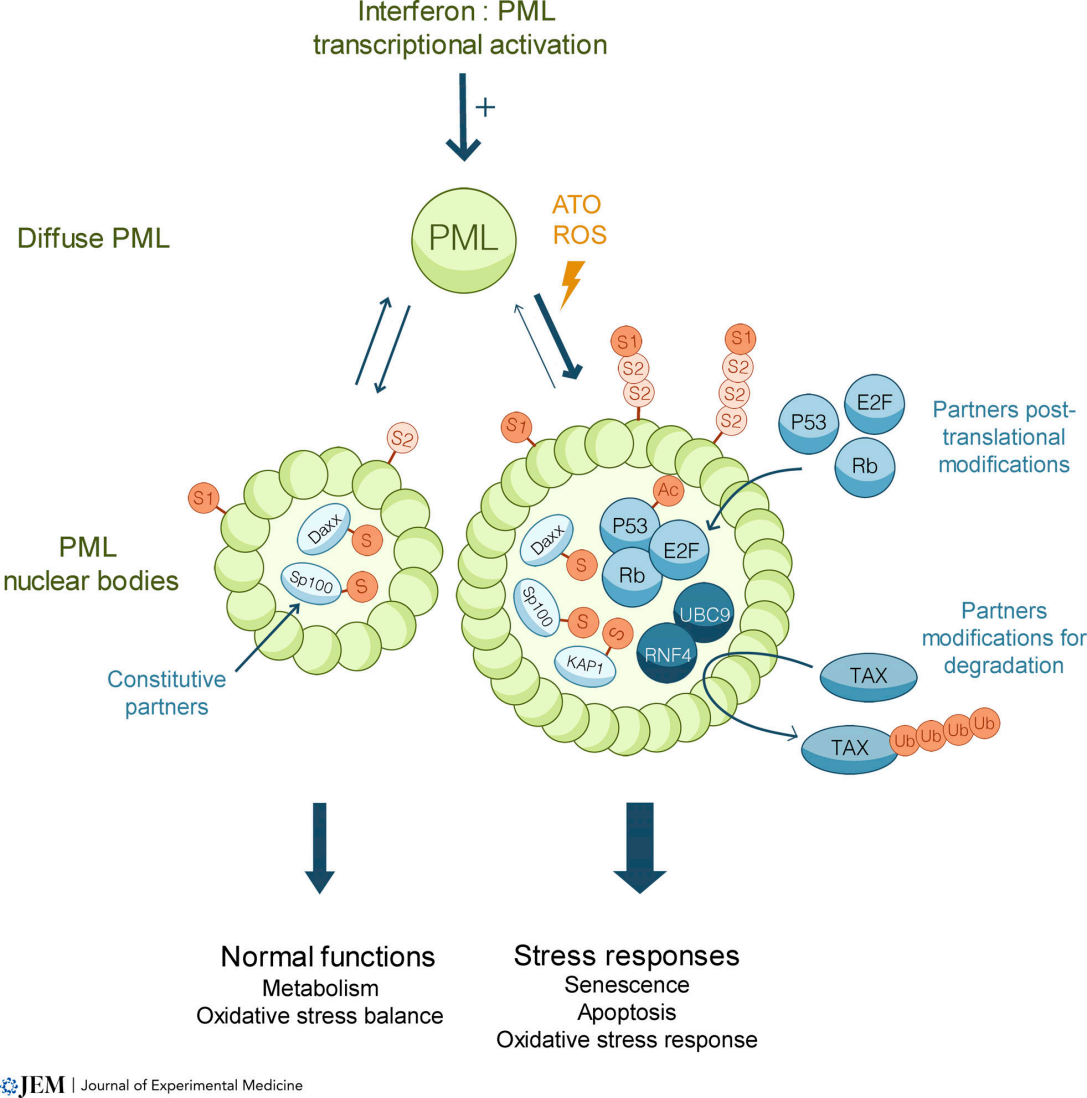
**PML senses stresses and initiates cellular responses.** Under physiological conditions, PML proteins assemble into PML NBs that recruit client proteins within their core, where they can be post-translationally modified, notably by SUMOs. Under oxidative stress or ATO treatment, diffuse nucleoplasmic PML rapidly aggregates into PML NBs. Stress-induced client recruitment within PML NBs, their modification and/or degradation, enhance cellular responses to stress, including senescence and apoptosis. S: SUMO, Ub: Ubiquitin, Ac: Acetylation.

PML belongs to the Tripartite Motif (TRIM or RBCC) family, characterized by a RING finger, one or two B-Boxes, and a coiled-coil domain, which are all required for PML oxidation, multimerization, and post-translational modifications. PML is expressed as a family of seven splice variants, all sharing the N-terminal RBCC core, but differing in their C-terminal parts (Condemine et al., 2006). Some isoforms have specific interactants and may consequently exert different functions when expressed individually, for example, on control of viral replication (Mai et al., 2022; Mathavarajah et al., 2023) or homologous recombination (Attwood et al., 2020; reviewed in Uggé et al., 2022). PML drives assembly of membrane-less nuclear domains named PML nuclear bodies (NBs). PML constitutes their external shell, and multiple client proteins can be recruited within the inner NBs core (Lallemand-Breitenbach and de The, 2018; Fig. 1). PML is very efficiently sumoylated and harbors a Small Ubiquitin-like MOdifier (SUMO) interacting motif (SIM). Contrasting with initial models (Müller et al., 1998; Shen et al., 2006), SUMO/SIM interactions are insufficient to promote the initial PML aggregation into NBs (Sahin et al., 2014b), which rather relies on PML oxidation (Jeanne et al., 2010). SUMO conjugation of PML K160 drives subsequent interactions with client proteins through their SIMs (Lallemand-Breitenbach et al., 2001; Sahin et al., 2014b). PML NBs’ association with some clients (such as Daxx or SP100) is “constitutive” (Ishov et al., 1999), while others are recruited to NBs under specific stress conditions, such as P53 and its modifying enzymes (Liebl and Hofmann, 2022; Vernier et al., 2011; Fig. 1). Only a variable fraction of PML is NB associated, some of the protein being also anchored onto chromatin, nuclear envelope, or even cytoplasm (Bellodi et al., 2006). PML bodies are dynamic structures that reversibly aggregate from the diffuse nucleoplasmic PML pool (Brand et al., 2010; Weidtkamp-Peters et al., 2008). Cell biology of PML NB biogenesis was enlightened by studies of PML interactions with ATO, which binds PML cysteines to promote NB assembly (Jeanne et al., 2010). This is followed by PML hypersumoylation, subsequent client recruitment, and client sumoylation. PML hypersumoylation later initiates PML degradation through the RNF4 SUMO-dependent ubiquitin ligase (Lallemand-Breitenbach et al., 2001, Lallemand-Breitenbach et al., 2008; Tessier et al., 2022; Zhu et al., 1997). The actual molecular details of how arsenic binding promotes NB assembly remain to be defined. Yet, mutations in PML arsenic binding site in Box B2 (discovered in therapy-resistant APL patients) alter basal NB assembly and blunt ATO response (Jeanne et al., 2010; Lehmann-Che et al., 2014; Liu et al., 2016; Zhu et al., 2014).

PML is an exquisitely oxidation-sensitive protein and a physiological sensor for ROS, which promote PML NB formation, similar to ATO (Guan et al., 2014; Guo et al., 2014b; Niwa-Kawakita et al., 2017; Sahin et al., 2014b). Importantly, PML was implicated in multiple forms of oxidative stress responses (Alhazmi et al., 2020; Niwa-Kawakita et al., 2017; Sahin et al., 2014b) and hypoxia signaling (Bernardi et al., 2006; Salsman et al., 2017; Yuan et al., 2011). P53 targets and the antioxidant response (NRF2 targets) are activated following acute oxidative stress in a PML-dependent manner (Guo et al., 2014b; Malloy et al., 2013; Niwa-Kawakita et al., 2017). *Pml*^*−/−*^ mice are normal but display unequivocal phenotypes when submitted to chemical stress, such as irradiation or high fat diet, which drives high levels of ROS or body weight increase (Carracedo et al., 2012; Niwa-Kawakita et al., 2017). Considering PML’s exquisite sensitivity to oxidation and its ability to trigger downstream antioxidant responses, basal oxidative stress derived from cell culture conditions (rarely conducted in 3% O_2_ atmosphere) or use of oxygen-adapted established cell lines rather than primary cells may have blurred results from some studies. It is thus important to focus on in vivo situations wherein endogenous PML proteins exert unambiguous phenotypes.

## Regulation of *PML* expression

*PML* expression is modulated by key stress and senescence pathways. First, transcriptionally, P53 and ARF (Alternative Reading Frame) dramatically upregulate *PML* expression (de Stanchina et al., 2004), a likely important feedforward mechanism in senescence induction (see below). *PML* transcription is also induced by IFN type I and II (Stadler et al., 1995; Fig. 1). PML plays a significant role in antiviral responses (Geoffroy and Chelbi-Alix, 2011; Patra and Müller, 2021) through both a direct interference with replication of multiple viruses, but also by enhancing global IFN response through enhancement of STAT1 signaling (Scherer and Stamminger, 2016). Interestingly, several PML NB client proteins are also IFN-inducible and modulate antiviral responses, including Sp100, Daxx, or SUMO (Grötzinger et al., 1996; Sahin et al., 2014a). Finally, estrogen signaling through estrogen receptor β transcriptionally induces *PML* gene expression, modifying Survivin and P21 expression through modulation of AKT (Datta et al., 2019).

Apart from transcriptional regulation of the gene, PML protein’s stability is finely tuned by multiple post-translational modifications. The first identified one is ATO-induced sumoylation, a consequence of ATO-driven NB formation, which promotes RNF4-mediated ubiquitination and degradation by the proteasome (Lallemand-Breitenbach et al., 2001, Lallemand-Breitenbach et al., 2008; Tatham et al., 2008; Zhu et al., 1997), defining a novel proteolytic pathway. PML can also be phosphorylated, acetylated, or ubiquitinylated, and many of these modifications were associated with modulation of PML stability or function (Hayakawa et al., 2008; Hayakawa and Privalsky, 2004; Shah et al., 2008). Several signaling pathways (notably kinases and de-ubiquitinase: CK2, USP11, USP7, etc.) converge onto PML degradation, sometimes downstream of identified oncogenes (Sarkari et al., 2011; Scaglioni et al., 2006; Wu et al., 2014; Yuan et al., 2011; reviewed in Gamell et al., 2014). Inhibition of these signaling pathways in tumor cells may thus promote PML NBs’ restoration.

## PML, a key senescence gene and tumor suppressor

Overexpression of mutant Ras in primary MEFs induces a senescence phenotype in *Pml* proficient, but not in *Pml*^*−/−*^ primary MEFs (Ferbeyre et al., 2000; Pearson et al., 2000). In fact, PML^−/−^ cells are profoundly resistant to senescence in multiple other experimental settings (Bernardi et al., 2008). PML can drive senescence at least in part through its ability to control P53 signaling. Interestingly, ex vivo, overexpression of a single PML isoform, PML-IV, can induce senescence by itself (Bischof et al., 2002) through a mechanism involving P53 and ARF (Ivanschitz et al., 2015). Yet, PML-IV has a low basal abundance when compared with PML-I, a protein that retains other ancestral domains (Mathavarajah et al., 2023). PML can also trigger P53-independent senescence pathways: in primary human fibroblasts, PML may colocalize with RB (Retinoblastoma) and E2F and trigger RB/E2F-dependent senescence (Mallette et al., 2004; Vernier et al., 2011).

Recent studies have demonstrated that PML NB formation promotes client sumoylation (Sahin et al., 2014b; Tessier et al., 2022), an important post-translational modification directly implicated in the control of senescence (Bischof and Dejean, 2007; Yates et al., 2008). This SUMO connection is a possible unifying mechanism underlying many effects of *PML* ablation or overexpression, since most of the numerous PML-sensitive pathways appear to be also strongly influenced by sumoylation of some of their key regulators (Fig. 1). However, the actual mechanistic links between PML, sumoylation, and senescence deserve more specific studies, notably in pathophysiological conditions, rather than on overexpression of PML-IV.

PML behaves as a weak tumor suppressor in vivo. Upon exposition to chemical tumor initiators, PML-null mice develop significantly more tumors than their wild-type counterparts (Wang et al., 1998), particularly in the presence of another activated oncogenic pathway (Haupt et al., 2013; Scaglioni et al., 2006; Trotman et al., 2006; Wolyniec et al., 2012). In a murine APL model in which *PML::RARA* is expressed under the cathepsin G promoter, PML loss decreases the time of APL onset (Rego et al., 2001). In a P53-mutated mouse model, PML loss still induced a reduction in overall survival and an increase in the number of tumors per mouse, arguing that PML exerts P53-independent roles in this setting (Haupt et al., 2013). In primary human tumor samples (for instance in carcinomas from various organs such as skin, breast, colon, lung, or prostate), PML expression and NB formation are initially increased upon transformation but lost when the cancer cells turn invasive (Gambacorta et al., 1996; Gurrieri et al., 2004; Koken et al., 1995), likely mirroring senescence/apoptosis in the natural history of human cancer development.

Some studies have also unraveled a tumor-promoting role for PML through control of stemness and metabolic rewiring. In chronic myeloid leukemia, PML promotes stemness in hematopoietic cells, favoring the maintenance of leukemia-initiating cells (Ito et al., 2008). Similarly, PML stimulates metabolic fueling (lipids, ATP) of cancer cells, and high PML expression correlates with poor prognosis in triple-negative breast cancers (Carracedo et al., 2012). This may relate to the fact that these breast tumors are almost always P53 mutants, so that the P53-dependent PML pro-senescent role is lost. In these situations where PML favors tumor maintenance, it could emerge as a relevant therapeutic target. While acute ATO exposure may be considered as an activation of PML-NB-dependent responses (notably acute enhancement of sumoylation), chronic ATO exposure, by promoting complete PML degradation, may ultimately inactivate PML-responsive pathways (Ito et al., 2008). Yet, ATO is toxic and protumor effects preclude its chronic use, so that novel PML degraders may be sought for these settings.

## PML in cancer cell clearance

**Figure 2.**
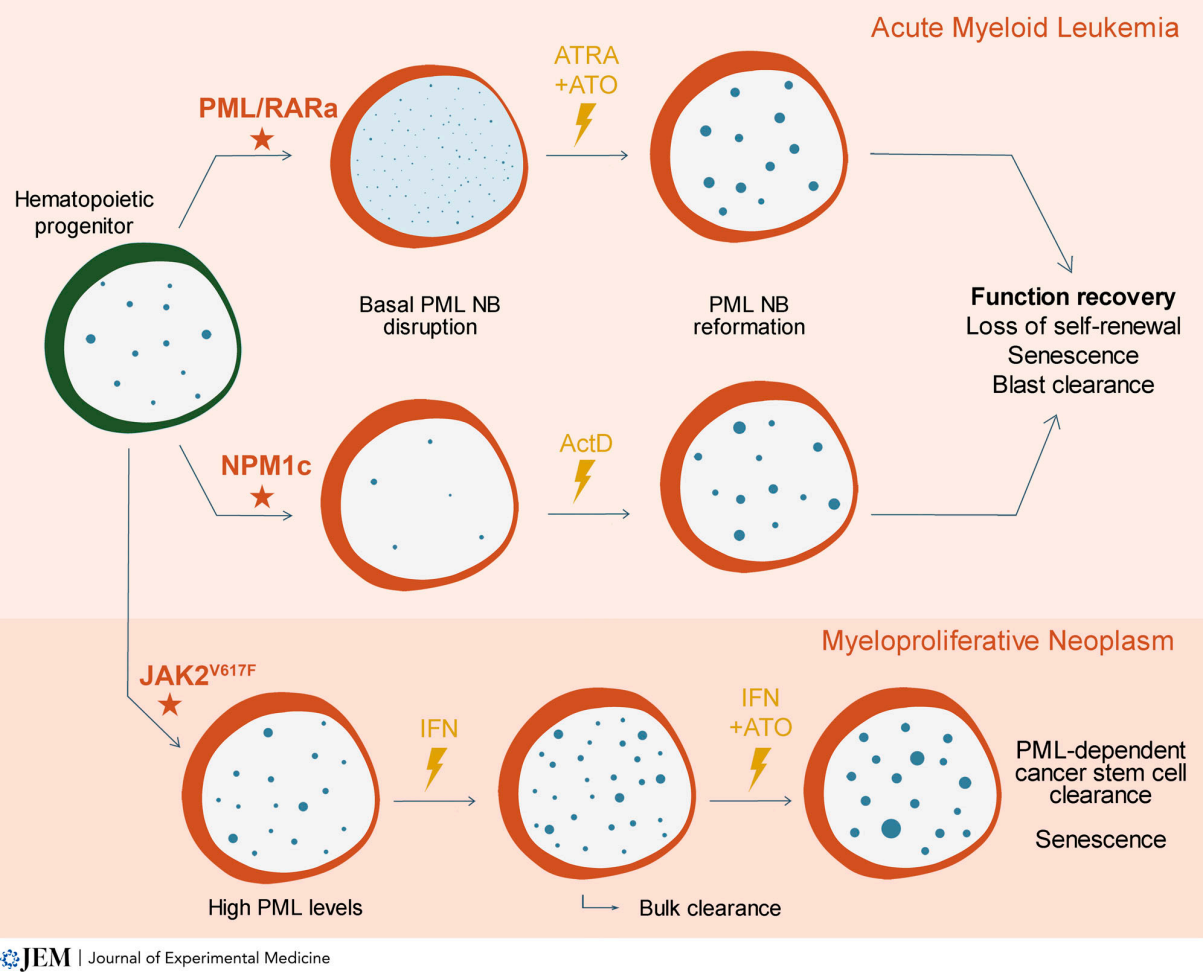
**Therapies activate PML-dependent cancer cell clearance in hematological malignancies.** Two subsets of AMLs (APL and NPM1c AML) exhibit basal impaired PML NB formation. Their reformation by oxidative stress–inducing therapies restores senescence and triggers blast clearance. In APL, PML is required for cure. In *JAK2*^*V617F*^-driven myeloproliferative neoplasms, IFN therapy is potentiated by ATO, their combination leading to *PML*-dependent cancer stem cell clearance. Blue dots represent PML nuclear bodies; red stars indicate oncogenic mutations in the hematopoietic progenitor; cancer therapies are signified by lightning symbols.

Cancer therapy is one of the most extreme forms of stress driving DNA damage or oxidative stresses, both implicated in therapy response (Gorrini et al., 2013). The key role of PML in therapy response was discovered in the context of *PML::RARA*-driven APL (Ablain et al., 2014). In APL, PML NB formation is impeded in the basal (untreated) state by large DNA-bound PML::RARA complexes that blunt NB assembly (Daniel et al., 1993; Koken et al., 1994). Such PML NBs disruption presumably impedes their normal functions. APL is cured by a combination of ATO and all-trans retinoic acid (ATRA), two drugs that induce PML::RARA degradation (de Thé et al., 2017). PML::RARA degradation restores NBs’ assembly from the PML proteins expressed from the normal allele, a process absolutely required for therapy response (Fig. 2). PML NBs then activate a P53 checkpoint with features of senescence, required for full therapy response. Accordingly, in the absence of PML or P53, ATRA therapy promotes differentiation, but does not significantly prolong survival (Ablain et al., 2014). Such an essential role of PML NB reformation in APL response and the ability of ATO to directly promote PML biogenesis (Lallemand-Breitenbach et al., 2001; Zhu et al., 1997) argues that ATO might have dual synergistic roles (PML::RARA degradation and direct enforcement of PML NB reformation). Critically, this hypothesis is strongly supported by the observations of mutations of the ATO binding site of PML, but not PML::RARA, in some therapy-resistant patients (Iaccarino et al., 2016; Lehmann-Che et al., 2014). Such dual activity of ATO likely explains the much more potent clinical activity of ATO in APL when compared with ATRA. This key observation raises the tantalizing prospect that ATO targeting of normal PML may have some clinical utility even in hematologic malignancies where the *PML* gene is not rearranged.

### Can PML targeting improve therapy response in non-APL leukemia?

In 30% of AML patients, nucleophosmin 1 (NPM1) frameshift mutations yield a de novo nuclear export signal that retargets this chaperone from the nucleolus to the cytoplasm (Falini et al., 2020). Unexpectedly, in AML cells bearing this NPM1 mutation (NPM1c), PML NBs are disorganized (El Hajj et al., 2015; Martelli et al., 2015) through a direct interference between NPM1c and PML (Wu et al., 2021). Since NPM1c weakens nucleolar organization, Actinomycin D (ActD), an inhibitor of RNA polymerase I activity disrupting nucleolar assembly, was proposed to exert synthetic lethal interactions with NPM1c. In NPM1c AML patients, pilot studies with single agent ActD showed unambiguous clinical activity and actually cured the index AML patient (Falini et al., 2015; Gionfriddo et al., 2021). Biologically, therapeutic concentrations of ActD rapidly poison mitochondria—most likely through mitochondrial DNA intercalation—to induce ROS production and acute oxidative stress which drive PML NBs reformation in NPM1c-expressing cell lines or patients in vivo (Wu et al., 2021). Downstream of PML NB reformation, ActD activates PML/P53-driven senescence and loss of clonogenic activity in NPM1c AML cell lines. Altogether, NPM1c-driven PML NB alteration, their reformation by therapy, and downstream PML/P53 dependent antitumor effects strikingly resemble the APL model (Fig. 2). They provide a proof of principle that PML may be a key therapeutic switch even in the absence of *PML* gene alteration.

Several studies have highlighted the importance of IFN signaling downstream of radio-, chemotherapy, or demethylating agents (Linnekamp et al., 2017; McLaughlin et al., 2020; Sistigu et al., 2014), all settings where IFN antitumor effects could involve PML and P53 (Kim et al., 2007). PML NBs are highly druggable: IFN exposure can boost PML protein levels, while ATO will subsequently enforce PML NB formation, their combination yielding large hyperactive PML NBs (Niwa-Kawakita et al., 2017; Quignon et al., 1998; Sahin et al., 2014b; Figs. 1 and 2). Myeloproliferative neoplasms bearing *JAK2*^*V617F*^ mutations are clinically sensitive to IFNα, which is the standard of care in Europe (Brkic and Meyer, 2020). In principle, one way to assess PML involvement is to look for any synergy with ATO. Indeed, ATO strongly potentiates the efficiency of IFN in a mouse model of *Jak2*^*V617F*^ myeloproliferative neoplasm, allowing leukemia-initiating cell clearance (Dagher et al., 2021; Fig. 2). Treating mice engrafted with a mix of *Pml*^*+/+*^ *Jak2*^*V617F*^ and *Pml*^*−/−*^*Jak2*^*V617F*^ cells by IFN/ATO led to a dramatic enrichment of *Pml*^*−/−*^*Jak2*^*V617F*^ cells, demonstrating the essential role of PML in the clearance process. Some evidence points to tumor stem cell senescence as the driver of PML-dependent cancer cell clearance.

Formation of large PML NBs by the IFN/ATO combination and promotion of client proteins sumoylation can also drive changes in client stability (Tessier et al., 2022). Indeed, PML NBs not only concentrate the enzymatic machinery for sumoylation but also for SUMO-initiated ubiquitination and degradation through RNF4 (Lallemand-Breitenbach et al., 2008). Thus, PML NBs may also promote client degradation, including that of oncoproteins (Lallemand-Breitenbach et al., 2008; Sahin et al., 2014b; Tatham et al., 2008). For example, the TAX viral oncoprotein drives adult T cell leukemia/lymphoma (Bazarbachi et al., 2011; Nasr R et al., 2003). The IFN/ATO combination drives apoptosis in these leukemic cell lines, clears the disease in mouse models, and has some clinical efficacy in patients (El Hajj et al., 2010; Kchour et al., 2009). Mechanistically, Tax bodies colocalize with PML NBs, and PML promotes Tax conjugation by SUMO2/3, leading to RNF4-dependent proteasomal degradation of the viral oncoprotein, a likely key contributor to therapy response (Dassouki et al., 2015; Fig. 1). Studies have also suggested that PML NBs can downregulate the abundance of misfolded proteins driving neurodegenerative conditions (Guo et al., 2014a). Overall, this ability of PML to broadly modulate protein stability (even apart from toxic or oncogenic proteins) may also mechanistically contribute to ROS or IFN responses and be therapeutically exploited.

### Through which cellular mechanisms could PML promote therapy response?

PML NBs can recruit P53 and its activating enzymes, virtually all of which may be SUMO-conjugated (Liebl and Hofmann, 2022; Matt and Hofmann, 2018). This stress-sensitive substrate/enzyme concentration should greatly enhance the efficiency of P53 post-translational modifications and subsequent transcriptional activation of senescence or apoptosis effectors. Thus, in this setting, PML NBs would promote recruitment/sumoylation of P53-modifying enzymes and enforce secondary P53 post-translational modifications that boost its signaling.

PML may also modulate senescence and therapy response through the global control of sumoylation. Interestingly, therapy-induced changes in sumoylation were tightly correlated to AML’s response to chemotherapy (Benoit et al., 2021; Bossis et al., 2014; Gâtel et al., 2020). Global inhibition of sumoylation has a favorable therapeutic impact, at least in part through activation of IFN signaling (Benoit et al., 2021; Lightcap et al., 2021; Nakamura et al., 2022). In that respect, PML not only promotes acute stress-induced sumoylation but may also regulate basal levels of this modification through RNF4-initiated degradation of SUMO conjugates (Sahin et al., 2014b; Tessier et al., 2022).

PML was proposed to bind TET2 and contribute to basal- or chemotherapy-induced changes in DNA methylation and gene expression (Song et al., 2018). While the consequences of these PML/TET2 enforced epigenetic changes were not explored, one should note that in APL, two master genes involved in leukemia initiation or progression (*DNMT3A* and *WT1*) regulate the status of 5-methylcytosine, similar to *TET2* (Cole et al., 2016; Lehmann-Che et al., 2018; Madan et al., 2016; Rampal et al., 2014; Wang et al., 2015; Zhao et al., 2019). A fraction of PML is chromatin-associated. In that respect, recent studies have demonstrated a key role of PML in epigenetic control of transposable element silencing in mouse embryonic stem cells through promotion of KAP1/TRIM28 complex sumoylation (Tessier et al., 2022). Future studies should explore whether epigenetic control impacts PML-modulated therapeutic response.

PML overexpression can dramatically sensitize cells to apoptosis. PML-null mice survive better than wild-type mice after irradiation or anti-Fas antibody (Bernardi et al., 2008). The mechanisms through which PML could regulate apoptosis may vary from one cellular system to another. Apart from PML/P53-dependent apoptosis, some groups have described PML-dependent caspase activation (Okazaki et al., 2012). For example, in multiple myeloma or hepatocellular carcinoma (Crowder et al., 2005; Herzer et al., 2009), TNF-related apoptosis-inducing ligand is upregulated in a PML-dependent manner. Moreover, PML can favor DNA damage responses that are upstream or independent from P53 in different models, for instance through *c-Jun* or *CHK2* (Dellaire et al., 2006; Salomoni et al., 2005; Yang et al., 2006).

### A PML/mitochondrial apoptotic or metabolic connection?

Complicating the elucidation of PML/apoptosis crosstalks, PML exerts a critical role on mitochondria, a key apoptosis regulator. PML may act upstream of peroxisome proliferator-activated receptors (PPARs) signaling, notably through the control of the acetylation status of its PGC1A co-activator. PPARs are a family of transcription factors sensing nutrients and modulating metabolism, in particular fatty acid oxidation. A PML–PPAR–fatty acid oxidation axis fuels asymmetric division and normal hematopoietic stem cell pool maintenance (Ito et al., 2012) or triple-negative breast cancer survival (Carracedo et al., 2012). This PML-controlled mitochondrial fitness could be tightly linked to therapeutic response (see below). In addition to NB-enforced PGC1A/PPAR activation, the abundantly expressed PML-I isoform contains a nuclear export signal, inducing its nucleo-cytoplasmic shuttling. This PML cytoplasmic fraction was involved in mitochondrial–endoplasmic reticulum contact sites, where it could participate in the transfer of calcium from the endoplasmic reticulum to mitochondria and confer sensitivity to death (Giorgi et al., 2010). Interestingly, cytoplasmic PML was also proposed to dump the autophagic flux and prevent uncontrolled growth (Missiroli et al., 2016). In AML models where the NPM1c mutation targets PML and NBs organization, major alterations in mitochondrial fitness were noted (Wu et al., 2021). The latter may account for the exquisite sensitivity of NPM1c AMLs not only to ActD but also to Venetoclax, a pro-apoptotic BCL-2 inhibitor (Masarova et al., 2021). Indeed, ActD dramatically synergizes with Venetoclax in vivo in a PML-dependent manner (Wu et al., 2021). Several studies focusing on SUMO-proteases have stressed the key role of SUMOs in the control of mitochondrial apoptosis (Fu et al., 2014; Guo et al., 2013; Prudent et al., 2015). Whether this is influenced by PML and may also contribute to the PML/mitochondria apoptosis axis is unsettled.

Mitochondria/drug sensitivity correlations appear to be quite specific for tumor/therapy pairs. In ovarian cancers, high levels of oxidative phosphorylation (OXPHOS) correlated with cis-platin response (Gentric et al., 2018). In this setting, high PML protein levels (but not transcript) were tightly correlated to OXPHOS status, stressing the role of post-translational modifications-regulated stability of endogenous PML proteins. In contrast, high mitochondrial OXPHOS activity actually predicts resistance to cytarabine in AML (Bosc et al., 2021; Farge et al., 2017). These opposing effects might be linked to *P53* status (ovarian cancers are almost always *P53* mutant, while the rare *P53*-mutant AMLs are constantly therapy resistant). How PML controls mitochondrial metabolic functions in basal condition or upon stress and any consequence for therapy response should be mechanistically approached through modulation of endogenous PML cytoplasmic localization. Finally, PML loss also induces mitochondrial defects and cytokine production in the microenvironment to boost tumor growth and impair therapy response (Missiroli et al., 2023). More broadly, possibly through the control of cytokine signaling, PML contributes to the cross-talk between the tumor and the microenvironment (Guarnerio et al., 2018) and could modulate response to immune therapies, as proposed for inhibitors of sumoylation (Lightcap et al., 2021).

## Concluding remarks

PML is a protein at the crossroads of multiple stress responses and acts as a sensor to amplify cellular responses that participate in cancer cell clearance, notably through control of SUMO conjugation. Multiple correlative studies in solid tumors have revealed tight (positive or negative) links between PML expression and patient outcomes. These may reflect intrinsic differences in tumor aggressiveness, P53 status, metabolism or interplay with their immune stroma, but also the ability of PML to arbitrate therapy response. The functional differences between PML isoforms, the respective importance of global PML abundance or its NB association, all call for a reappraisal of PML role in patient prognosis or drug response. Structure-function analysis of endogenous PML in tumor models in vivo could also provide invaluable mechanistic insights into therapy response. Finally, PML NBs are druggable, but could also be fine-tuned by other compounds than IFN or ATO, including drugs that specifically promote or impede NB biogenesis. PML-targeted pharmacology may only be in its infancy and greatly broadens the well-explored setting of APL therapy.
